# Prospective and longitudinal evolution of postoperative periprosthetic findings on metal artifact–reduced MR imaging in asymptomatic patients after uncemented total hip arthroplasty

**DOI:** 10.1007/s00256-020-03666-8

**Published:** 2020-11-09

**Authors:** Christoph Germann, Lukas Filli, Pia M. Jungmann, Dimitri N. Graf, Jan Fritz, Christian W. A. Pfirrmann, Reto Sutter

**Affiliations:** 1 Radiology, Balgrist University Hospital, University of Zurich, Forchstrasse 340, CH-8008 Zurich, Switzerland; 2 Department of Diagnostic and Interventional Radiology, Medical Center - University of Freiburg, Hugstetter Strasse 55, 79106 Freiburg, Germany; 3 Department of Radiology, NYU Langone Health, 660 1st Ave, New York, NY 10016 USA

**Keywords:** Hip, Total hip arthroplasty, Magnetic resonance imaging, Bone, Normal postoperative findings

## Abstract

**Objective:**

To prospectively assess the evolution of postoperative MRI findings in asymptomatic patients after total hip arthroplasty (THA) over 24 months (mo).

**Methods:**

This prospective cohort study included 9 asymptomatic patients (56.7 ± 15.0 years) after THA. Metal artifact–reduced 1.5-T MRI was performed at 3, 6, 12, and 24 mo after surgery. The femoral stem and acetabular cup were assessed by two readers for bone marrow edema (BME), periprosthetic bone resorption, and periosteal edema in addition to periarticular soft tissue edema and joint effusion.

**Results:**

BME was common around the femoral stem in all Gruen zones after 3 mo (range: 50–100%) and 6 mo (range: 33–100%) and in the acetabulum in DeLee and Charnley zone II after 3 mo (100%) and 6 mo (33%). BME decreased substantially after 12 mo (range: 0–78%) and 24 mo (range: 0–50%), may however persist in particular in Gruen zones 1 + 7. Periosteal edema along the stem was common 3 mo postoperatively (range: 63–75%) and rare after 24 mo: 13% only in Gruen zones 2 and 5. Twelve months and 24 mo postoperatively, periprosthetic bone resorption was occasionally present around the femoral stem (range: 11–33% and 13–38%, respectively). Soft tissue edema occurred exclusively along the surgical access route after 3 mo (100%) and 6 mo (89%) and never at 12 mo or 24 mo (0%).

**Conclusion:**

Around the femoral stem, BME (33–100%) and periosteal edema (0–75%) are common until 6 mo after THA, decreasing substantially in the following period, may however persist up to 24 mo (BME: 0–50%; periosteal edema: 0–13%) in few non-adjoining Gruen zones. Soft tissue edema along the surgical access route should have disappeared 12 mo after surgery.

## Introduction

The number of patients undergoing hip replacement surgery has drastically increased over the last decades [1]. Despite being one of the surgical procedures with the best outcome, complications such as aseptic loosening, periprosthetic fracture, hardware failure, wear-induced osteolysis/synovitis, and infection occur on a regular basis [2–4]. In order to improve patient outcomes, detecting these complications as early as possible is a major concern [5].

Radiographs represent the standard first-line imaging modality after total hip arthroplasty [6]. However, some alterations in the peri-implant region, e.g., in the periprosthetic bone marrow or soft tissues, may be the first signs indicating the abovementioned postoperative complications and cannot be depicted on radiographs [7].

Being the most accurate imaging modality to evaluate periprosthetic soft tissues, metal artifact–reduced MRI is of paramount importance to assess local complications after THA [2, 8–13]. Recently developed metal artifact reduction techniques such as Slice Encoding for Metal Artifact Correction (SEMAC) improve the detection of complications after THA [14–18] and advanced reconstruction techniques such as Compressed Sensing for SEMAC (CS-SEMAC) allow reducing the formerly long acquisition times [7, 19, 20]. These developments have sparked a new era in the visualization of the peri-implant region [1, 21].

However, the interpretation of MR images after THA remains challenging because there is substantial overlap between normal findings in asymptomatic patients and clinically relevant abnormal imaging findings. For example, periprosthetic bone marrow edema (BME) pattern—defined as the focal area of hyperintense intraosseous signal on fluid-sensitive sequences [22, 23]—is frequently encountered in asymptomatic individuals 1 year after THA [24]. Other peri-implant findings that are well depicted at MRI are (a) periprosthetic bone resorption, represented by a well-demarcated linear intraosseous band adjacent to the implant surface with high signal intensity on fluid-sensitive sequences, surrounded by a thin linear layer with low signal intensity [22]; (b) periosteal edema, defined as linear hyperintensity along the periosteum on fluid-sensitive sequences; and (c) periarticular soft tissue edema—depicted as a focal area of high signal intensity on fluid-sensitive sequences in the periprosthetic soft tissues (e.g., muscles). Thus far, there is no study available that addresses the chronological evolution of normal postoperative MR imaging findings after THA.

Therefore, the aim of this study was to prospectively evaluate the frequency of various MRI findings in the peri-implant region in asymptomatic patients following primary THA for a period of 24 months.

## Materials and methods

### Study population

This prospective single-center cohort study was approved by our local ethics committee. Written informed consent was given from all included subjects. Eleven of 175 potentially eligible consecutive patients agreed to participate in the study and were enrolled. Each of the potentially eligible patients underwent uncemented primary THA between April 2017 and October 2017 at our hospital. Inclusion criteria comprised: (a) age ≥ 18 years, (b) Western Ontario and McMaster Universities Arthritis Index (WOMAC) score ≤ 1 (considered asymptomatic) [25, 26], and (c) oral and written informed consent to participate in the study.

**Fig. 1:**
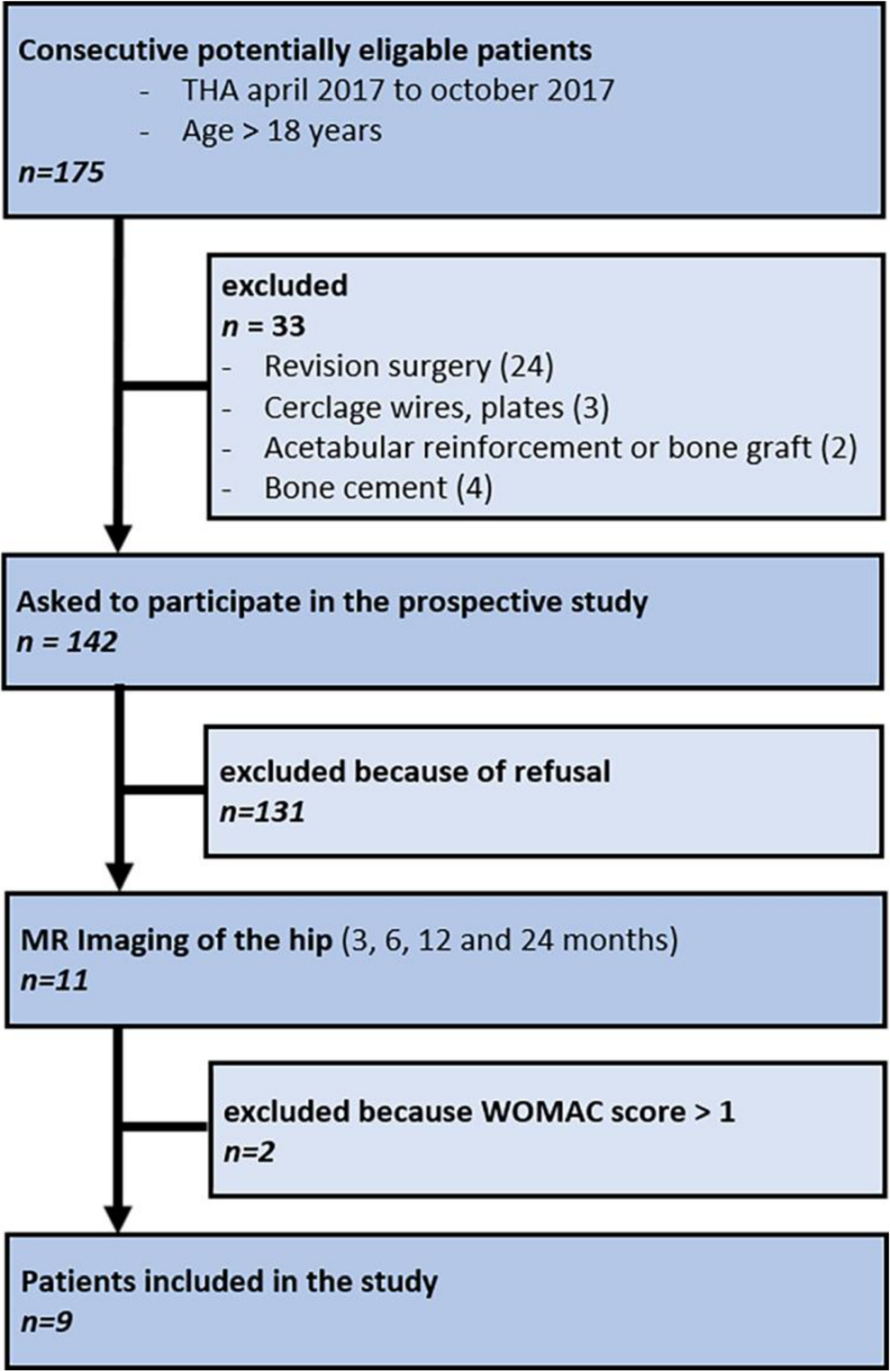
Flowchart of patient inclusion and exclusion. *THA*, total hip arthroplasty; *WOMAC*, Western Ontario and McMaster Universities Arthritis Index questionnaire

Exclusion criteria were as follows: (a) general contraindications for MRI (e.g., cardiac pacemaker) and (b) revision hip surgery or complex surgery (e.g., cerclage wires, acetabular reinforcement or bone grafting, cement and/or tumor prosthesis). The flowchart of patient inclusion and exclusion is presented in Fig. 1.

All prostheses were cementless systems with screwless acetabular cups and polyethylene inlays. The following implants were used: Versafitcup® and Quadra® system using a titanium-niobium alloy for the stem and a cobalt-chromium alloy for the femoral head (*n = 5*; Medacta); April Cup and SPS Evolution® Stem System using a titanium alloy for the stem and a cobalt-chromium alloy for the femoral head (*n = 1*; SymBios); and Fitmore® using a titanium alloy for the femoral stem and either a ceramic head or a cobalt-chromium alloy for the head (*n = 3*; Zimmer Biomet). The surgical approach was exclusively anterior; the capsulotomy was re-approximated and closed with dedicated sutures. Two board-certified hip-subspecialized orthopedic surgeons performed the procedure (surgeon 1 performed 3 of 9 arthroplasties and has 14 years of experience after board exam; surgeon 2 performed 6 of 9 arthroplasties with 6 years of experience after board exam). During the 24-month follow-up, none of the included subjects had revision hip surgery.

### Imaging

Each patient had a total of 4 MRI scans of the respective hip joint during the first 2 years following hip replacement (3, 6, 12, and 24 months, respectively). All MR images were acquired with a 1.5-Tesla MAGNETOM Avanto Fit system (Siemens Healthcare, Erlangen, Germany), using a combination of an 18-channel surface coil and a 32-channel spine coil. The same clinical MRI protocol was used in all cases—dedicated and optimized for metal artifact reduction around the hip implant. The protocol comprised a coronal STIR CS-SEMAC sequence as part of a vendor-specific work-in-progress package (Siemens Healthcare), an axial short τ inversion recovery (STIR) sequence with optimized inversion pulse [27], and high-bandwidth sequences in all standard imaging planes. The CS-SEMAC sequence was applied with 19 slice-encoding steps (SES), 10 iterations, and a normalization factor of 0.001 to achieve optimal image quality. Detailed imaging parameters are listed in Table 1.

**Table 1:** Detailed MRI protocol optimized for metal artifact reduction. *CS*, compressed sensing; *ETL*, echo train length; *FOV*, field of view; *NSA*, number of signal averages; *SEMAC*, slice encoding for metal artifact correction; *STIR*, shirt τ inversion recovery; *TA*, acquisition time; *TE*, echo time; *TR*, repetition time. Axial STIR* with optimized inversion pulse

Parameter	Coronal STIR SEMAC CS	Axial STIR*	Coronal T2 high bandwidth	Axial T1 high bandwidth	Sagittal T1 high bandwidth
TR/TE (ms)	4220/36	4000/31	4000/58	669/8.6	6277.3
ETL	9	11	15	3	3
NSA	1	3	2	2	2
Number of slices	25	27	20	29	31
Section thickness (mm)	4	7	4	6	4
Spacing (mm)	4	8.75	6	8.4	4.4
Matrix	256 × 205	384 × 269	512 × 282	512 × 410	320 × 320
FOV (mm^2^)	280 × 280	189 × 189	220 × 220	210 × 210	200 × 200
Bandwidth (Hz/pixel)	500	450	390	425	435
Slice-encoding steps	19	–	–	–	–
TA (min:s)	06:19	03:56	02:28	02:17	01:59

### MRI interpretation

All MR images were evaluated separately by two board-certified musculoskeletal radiologists (C. G and D.G., with 6 and 7 years of experience, respectively). Image analysis was performed in an independent and randomized fashion on anonymized data sets on a state-of-the-art picture archiving and communication system (Merlin, Phönix-PACS, Freiburg, Germany) workstation.

Each radiologist assessed the various Gruen zones and DeLee and Charnley zones [28, 29] with regard to the presence of periprosthetic bone marrow edema (BME) pattern, periprosthetic bone resorption, and periosteal edema using a binary scale (feature either present or absent). In order to be classified as a true finding, BME and periosteal edema had to be visualized in two imaging planes in the STIR sequences (axial and coronal) in the exact same location. We acknowledge the fact that in the radiological literature, the term “BME” is often used as a concise descriptive term on imaging, despite not being identical to the histopathologic diagnosis of bone marrow edema; however, in order to facilitate legibility of the manuscript, we use the term “BME” rather than “edema-like marrow signal intensity” or “bone marrow edema–like signal intensity” [30]. The presence of joint fluid was evaluated in a semiquantitative manner based on the maximum diameter of the fluid (“no to mild,” < 3 mm; “moderate,” 3–6 mm; “substantial,” > 6 mm) in four directions relative to the prosthesis (lateral, medial, anterior, and posterior, respectively). Moreover, the integrity of the hip abductor tendons (gluteus medius and gluteus minimus) was evaluated as either “intact” or “torn”: an intact tendon was defined as a homogenously low signal intensity continuous tendon without caliber irregularities in all sequences. Tendon discontinuity, intrasubstance high signal intensity on fluid-sensitive sequences, and/or fluid interposition between the tendon and osseous insertion were regarded as signs for a tendon tear.

### Statistical analysis

Statistical analysis was performed using SPSS (v25, IBM Corp., Somers, NY). General descriptive statistics were applied. The inter-reader agreement was determined by calculating Cohen’s *κ* for qualitative variables. Cohen’s *κ* was interpreted according to Kundel and Polansky as either “slight” (0–0.20), “fair” (0.21–0.40), “moderate” (0.41–0.60), “substantial” (0.61–0.80), or “almost perfect” agreement (0.81–1.00) [31]. Qualitative variables are presented as frequencies (percentages) for each time point.

## Results

### Study population

Eleven patients agreed to participate in the study (6 males, 5 females; mean age: 56.6 ± 14.8 years, range 22–70 years). Each subject had a total of 4 MRI scans during the 2 first years after THA: 3, 6, 12, and 24 months, respectively. All MRI examinations were completed successfully. After excluding every patient with a WOMAC score > 1 at each study visit (2 patients with a consecutive WOMAC score > 1 at each time point, respectively), a total of 9 patients were included (4 males, 5 females; mean age, 56.7 ± 15.0 years; age range 22–70 years). Due to missing data (no WOMAC score), one additional patient was excluded for the 3-month and 24-month MRI scan, respectively. WOMAC scores were as follows: 4.8 ± 1.5 (range 2.6–6.7) preoperatively; 0.6 ± 0.4 (range 0.0–1.0) at 3 months; 0.3 ± 0.4 (range 0.0–1.0) at 6 months; 0.3 ± 0.4 (range 0.0–1.0) at 12 months; and 0.2 ± 0.2 (range 0.0–0.5) at 24 months, respectively. Two of nine included patients (22%) were diagnosed with secondary osteoarthritis (one caused by femoroacetabular impingement, one by developmental dysplasia of the hip); the remaining seven of nine included patients (78%) were diagnosed with primary osteoarthritis.

### Inter-observer agreement

Inter-observer agreement was “substantial” for bone marrow edema pattern (*κ* = 0.71), periosteal edema (*κ* = 0.80), and joint fluid (*κ* = 0.75), and “almost perfect” for periprosthetic bone resorption (*κ* = 0.93) as well as periarticular soft tissue edema (*κ* = 0.91).

### Frequency of MRI findings

**Fig. 2:**
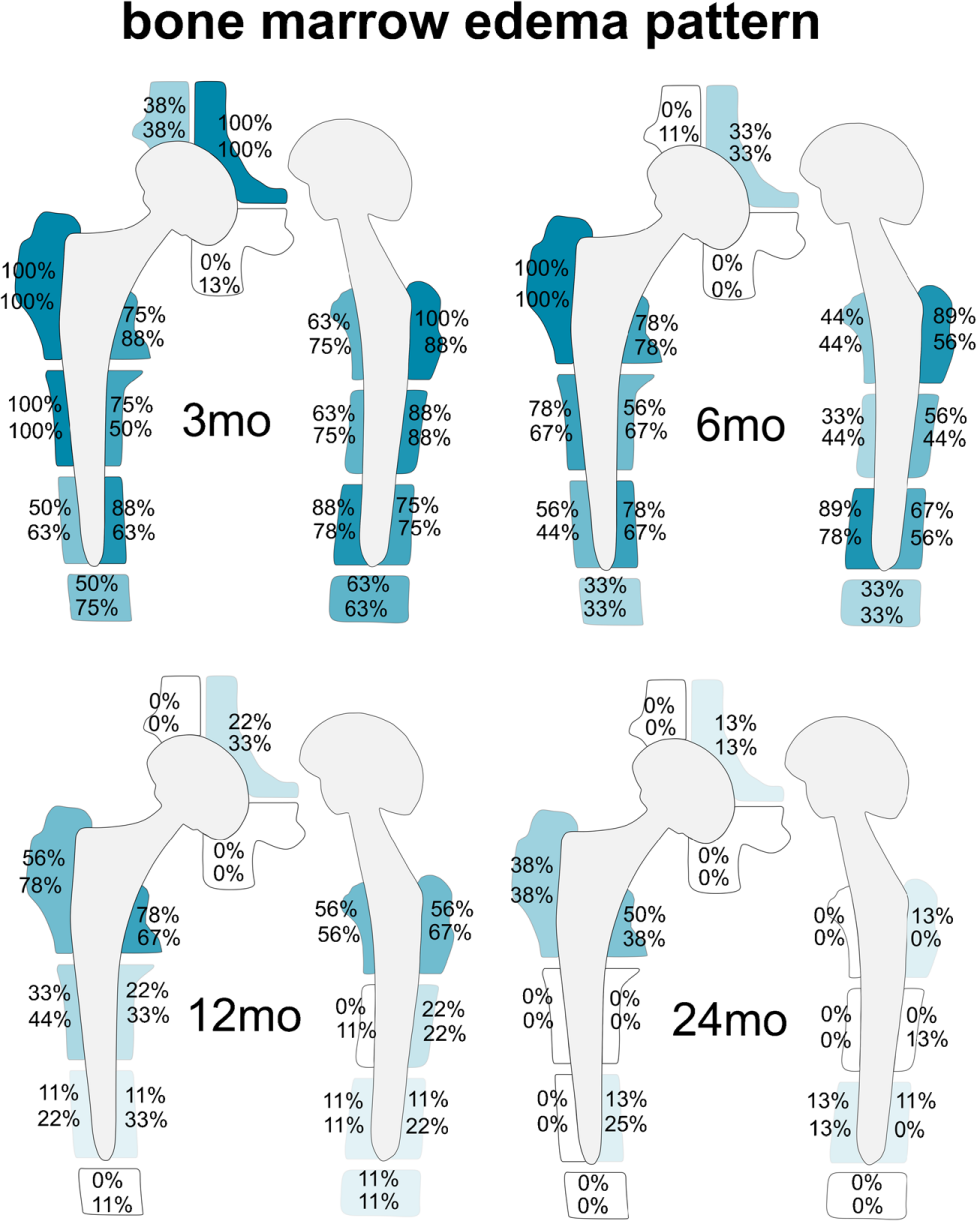
Schematic illustration for the frequencies of bone marrow edema pattern: for each zone, the upper number represents results for reader 1, the lower number for reader 2. The frequencies are indicated by different color intensities (darker color equals higher percentage and vice versa) and listed as percentages. Bone marrow edema pattern was a frequent finding around the femoral stem both 3 mo (range: 50–100%) and 6 mo (range: 33–100%) after THA, with a substantial decrease in the second postoperative year (12 mo range: 0–78%; 24 mo range: 0–50% for reader 1 and 0–38% for reader 2). 24 mo after surgery, bone marrow edema pattern persisted most commonly in Gruen zones 1 and 7. *mo*, months

**Fig. 3:**
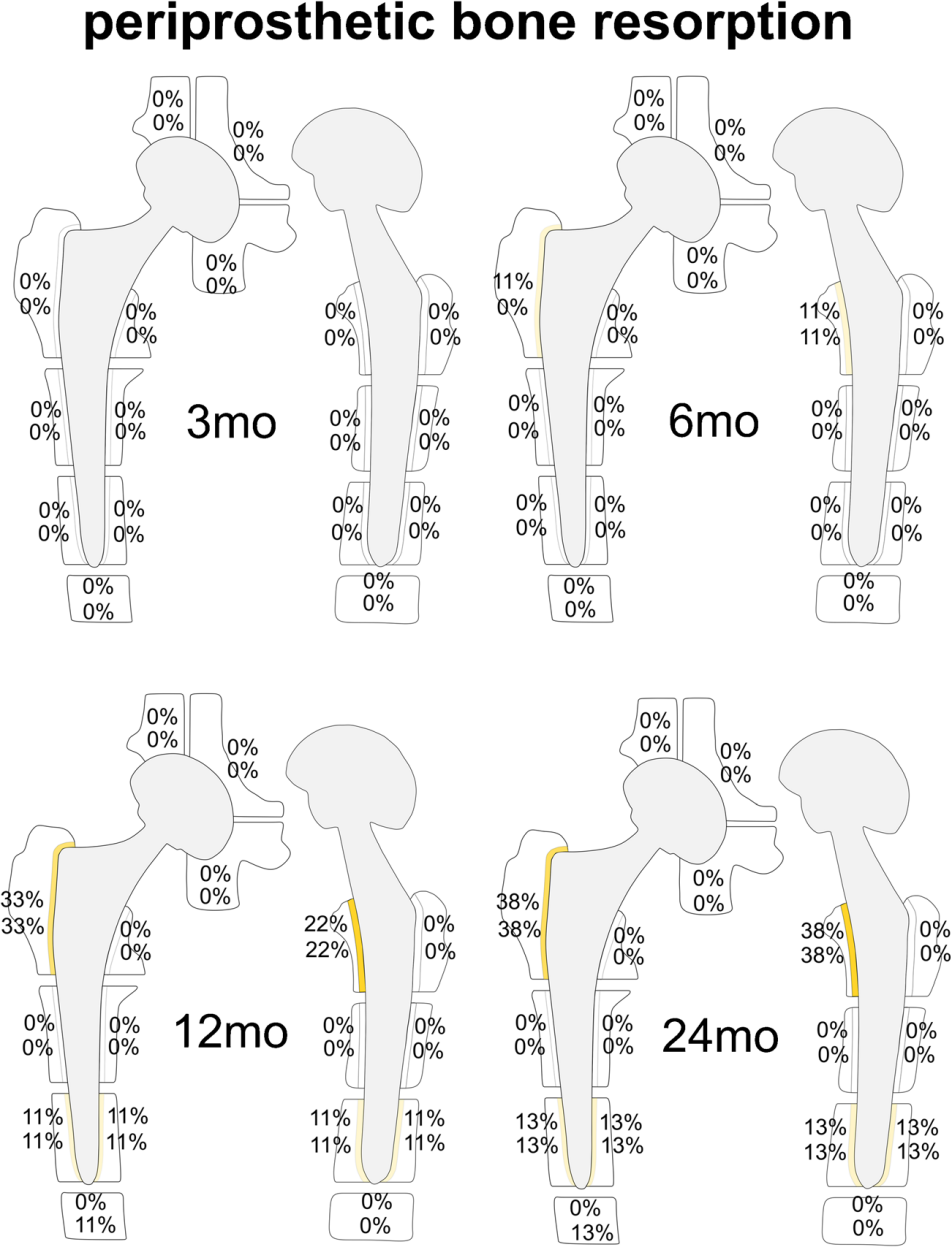
Schematic illustration for the frequencies of periprosthetic bone resorption: for each zone, the upper number represents results for reader 1, the lower number for reader 2. The frequencies are indicated by different color intensities (darker color equals higher percentage and vice versa) and listed as percentages. Periprosthetic bone resorption was most commonly encountered in Gruen zones 1 and 8, starting already 6 mo after THA in one participant (11%). Over time, there was a slight increase in periprosthetic bone resorption around the femoral stem (12 mo range: 0–33%; 24 mo range: 0–38%). *mo*, months; *THA*, total hip arthroplasty

**Fig. 4:**
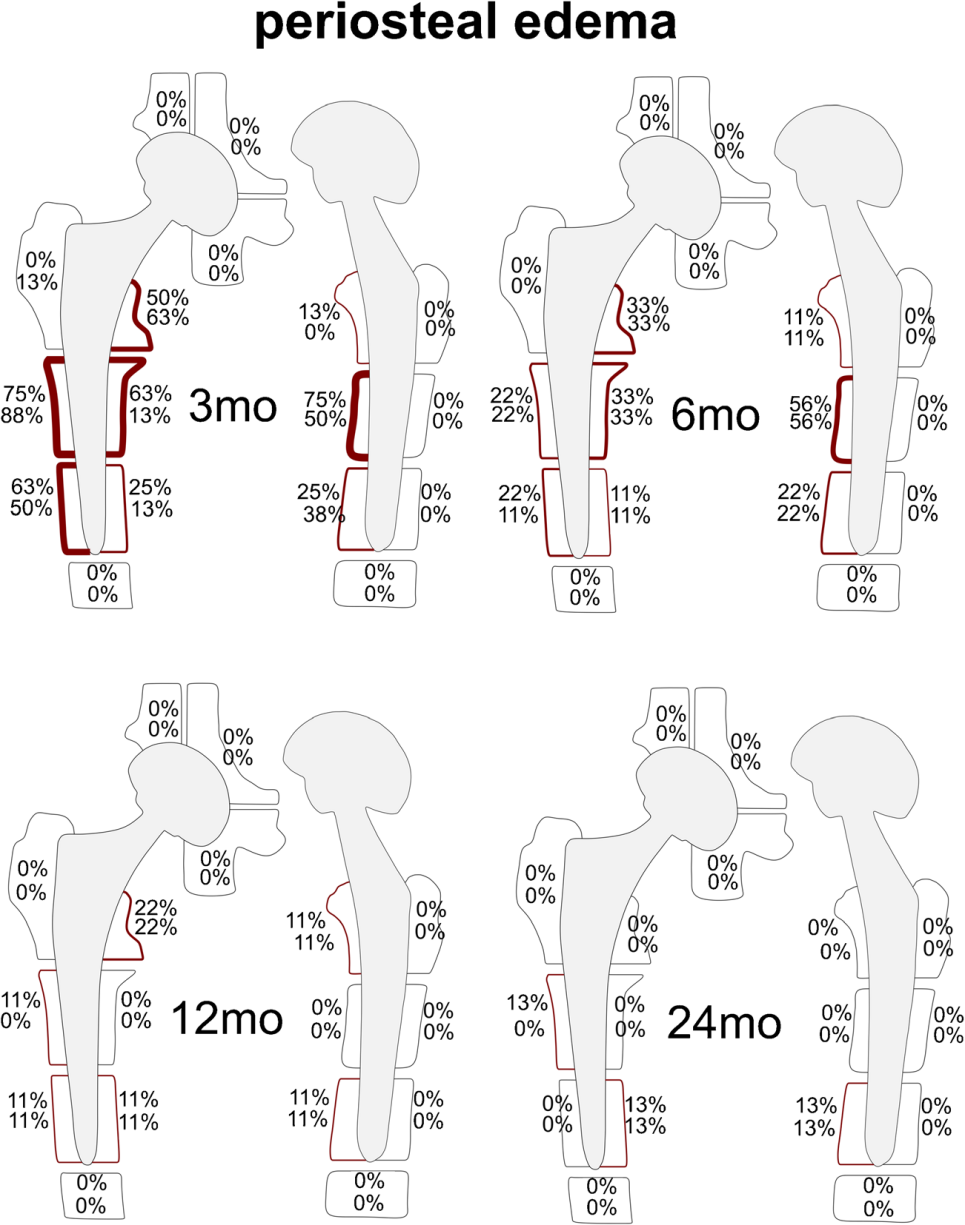
Schematic illustration for the frequencies of periosteal edema: for each zone, the upper number represents results for reader 1, the lower number for reader 2. The frequencies are indicated by different color intensities (darker color equals higher percentage and vice versa) and listed as percentages. Periosteal edema was a frequent finding around the femoral stem 3 mo after THA (range: 0–75% for reader 1 and 0–88% for reader 2), accentuated in Gruen zones 2, 3, 6, and 9. Over time, there was a substantial decrease around the femoral stem (12 mo range: 0–22%; 24 mo range: 0–13%) 24 mo after surgery periosteal edema persisted only in one patient in Gruen zones 2, 5, and 10, respectively. *mo*, months; *THA*, total hip arthroplasty

Detailed results and graphical illustrations for all Gruen zones and DeLee and Charnley zones regarding BME pattern, periprosthetic bone resorption, and periosteal edema are presented in Figs. 2, 3, and 4, respectively (reader 1).

#### Bone marrow edema

**Fig. 5:**
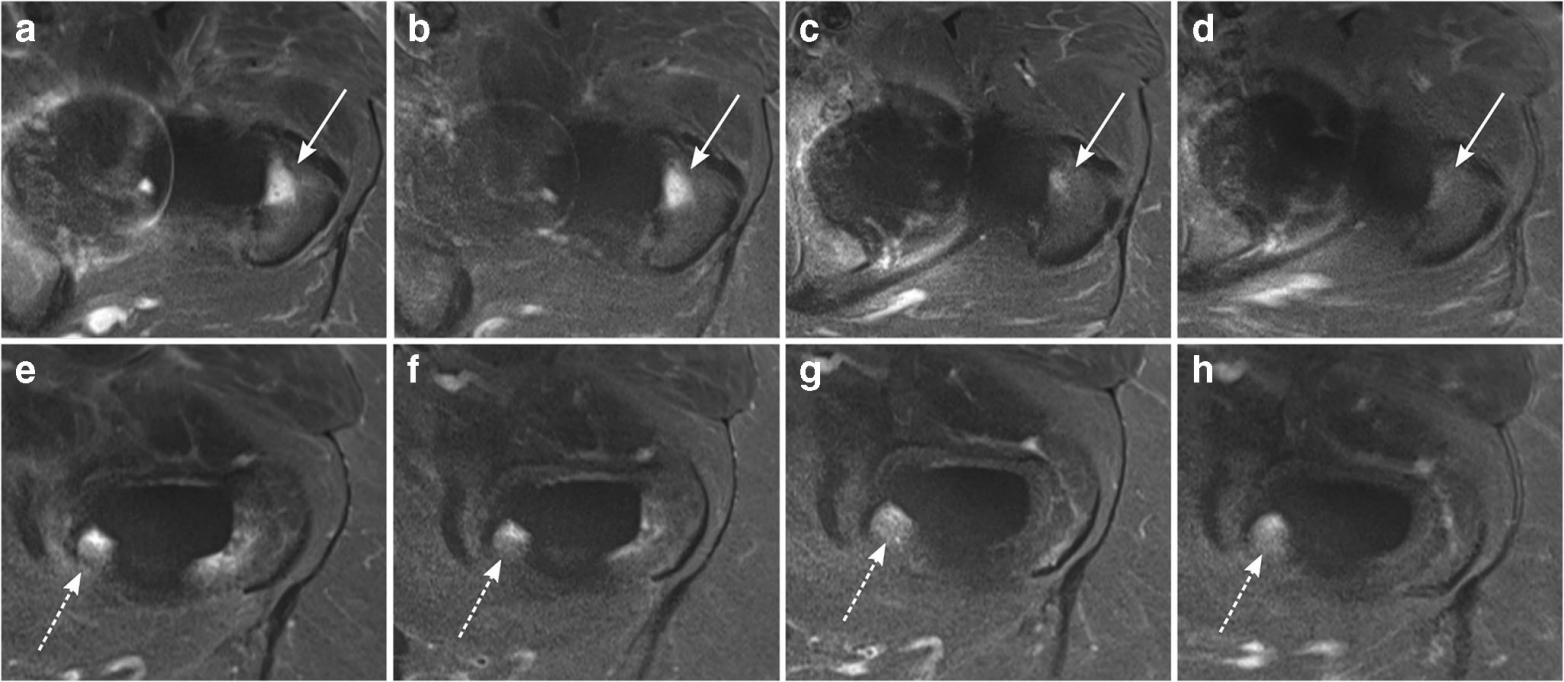
BME around the femoral stem on axial STIR MRI. A 68-year-old asymptomatic woman with left-sided THA and BME pattern around the femoral stem in Gruen zone 1 (arrows) decreasing over time (**a** = 3 months, **b** = 6 months, **c** = 12 months, and **d** = 24 months after THA). Persisting BME in the same patient in Gruen zone 7 (dashed arrows; **e** = 3 months, **f** = 6 months, **g** = 12 months, and **h** = 24 months after THA). *BME*, bone marrow edema; *THA*, total hip arthroplasty

BME pattern was a common finding around the femoral stem 3 and 6 months after surgery, most frequently in Gruen zones 1, 2, 5, 6, 7, 10, 12, 13, and 14 (3 months: range 75–100% for reader 1 and 63–100% for reader 2; 6 months: range 56–100% for reader 1 and 44–100% for reader 2). BME adjacent to the acetabular cup was present consistently after 3 months in DeLee and Charnley zone II for both readers (100%, respectively). In general, the extent of BME pattern decreased substantially with time after surgery; 12 months: range 0–78% for both readers; and 24 months: range 0–56% for reader 1 and range 0–38% for reader 2. BME persisted up to 24 months in various areas around the femoral stem, in particular in Gruen zones 1 and 7 (38% and 50%, respectively for reader 1; 38% and 38%, respectively for reader 2) (Fig. 5), whereas BME disappeared completely after 24 months in Gruen zones 2, 3, 4, 6, 8, 9, 11, and 12 (0% for both readers). Furthermore, there was no BME after 24 months in DeLee and Charnley zones I and III (0% for both readers).

#### Periprosthetic bone resorption

**Fig. 6:**
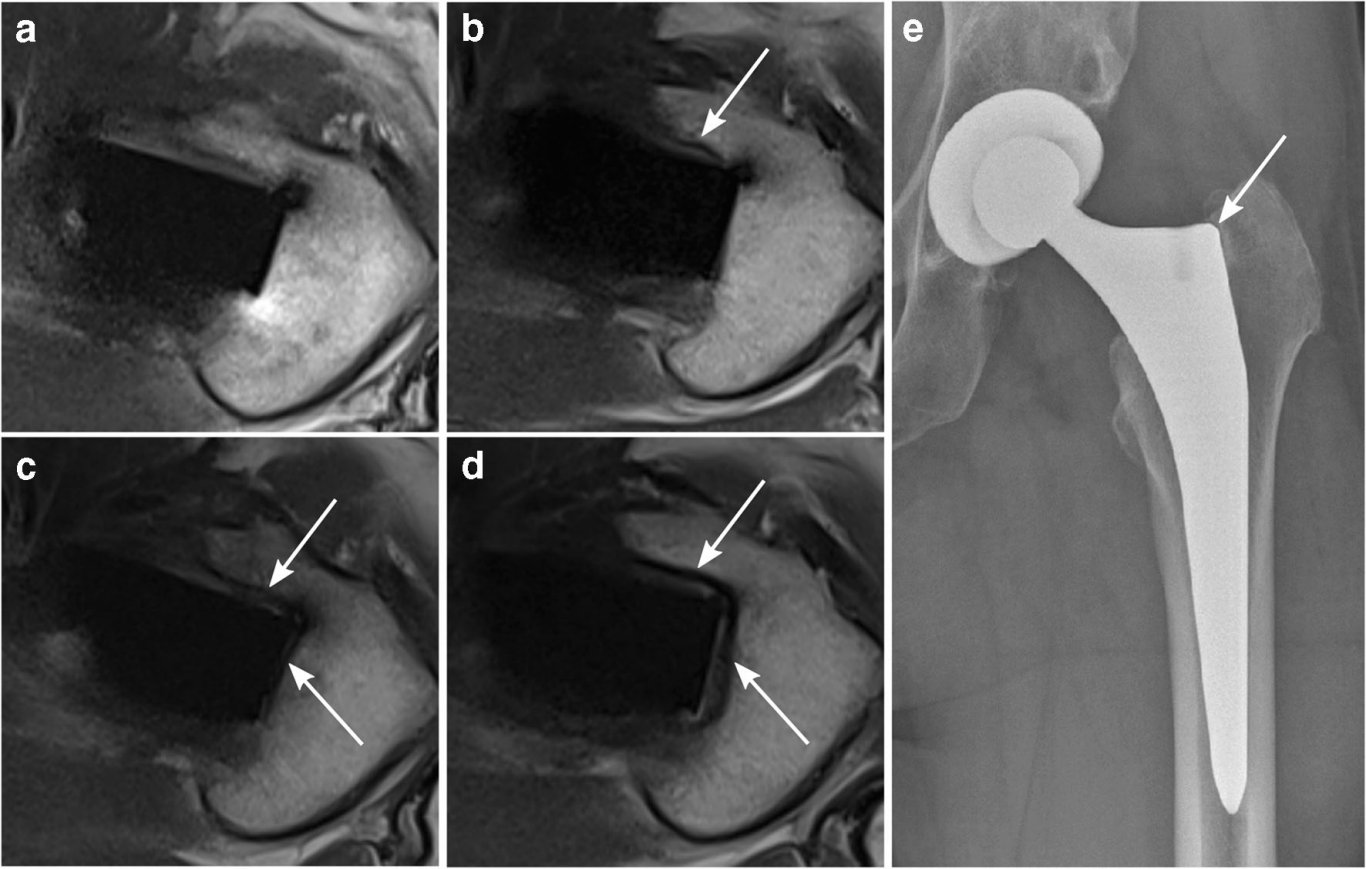
Periprosthetic bone resorption on axial T1w MRI. A 52-year-old asymptomatic woman with left-sided THA. A well-demarcated periprosthetic resorption zone (maximum width 2 mm) with a hypointense peripheral lining (arrows) is present laterally along the proximal femoral shaft (Gruen zone 1) 12 months (**c**) and 24 months (**d**) after surgery with no correlate 3 months (**a**) and 6 months (**b**) postoperatively. Additionally, a small resorption zone can be seen anteriorly along the proximal femoral shaft in Gruen zone 8 after 6, 12, and 24 months (**b**–**d**) without a definite correlate as early as 3 months (**a**). AP radiographic correlation 12 months after surgery (**e**) shows a demarcated narrow osteolytic zone in Gruen zone 1. *THA*, total hip arthroplasty

For both readers, periprosthetic bone resorption was present most commonly in Gruen zones 1 and 8 after 12 and 24 months: Gruen zone 1 for both readers: 33% after 12 months and 38% after 24 months, and Gruen zone 8 for both readers: 22% after 12 months and 38% after 24 months. Reader 1 described periprosthetic bone resorption in Gruen zones 1 and 8 for one patient (11%) already 6 months after surgery (Fig. 6). Both readers described periprosthetic bone resorption for one patient in Gruen zones 3, 5, 10, and 12 after 12 months (11%) and 24 months (13%), respectively. Additionally, reader 2 found periprosthetic bone resorption present in Gruen zone 4 in one patient after 12 months (11%) and 24 months (13%), respectively. The maximum width of the periprosthetic bone resorption was 2 mm.

#### Periosteal edema

**Fig. 7:**
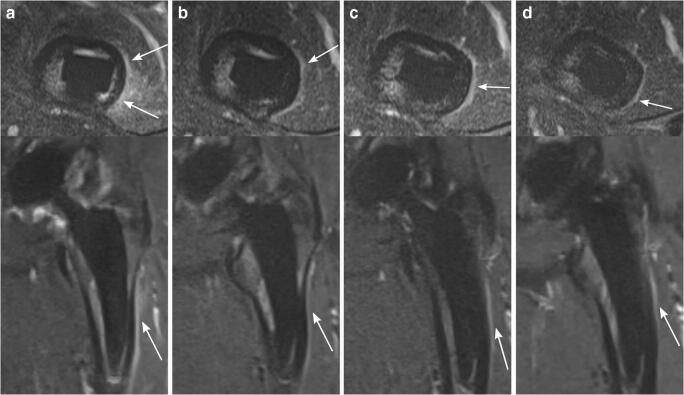
Periosteal edema on axial STIR MRI (upper row) and coronal STIR CS-SEMAC MRI (lower row). A 60-year-old asymptomatic woman with left-sided THA. Periosteal edema is present along the lateral side of the femoral shaft, accentuated in Gruen zone 2 (arrows), which persists over time. **a** = 3 months, **b** = 6 months, **c** = 12 months, and **d** = 24 months. *CS*, compressed sensing; *SEMAC*, slice encoding for metal artifact correction; *THA*, total hip arthroplasty

Three months after surgery, periosteal edema was frequently seen in Gruen zone 2 (75% for reader 1; 88% for reader 2), Gruen zone 3 (63% for reader 1; 50% for reader 2), Gruen zone 6 (63% for reader 1; 50% for reader 2), Gruen zone 7 (50% for reader 1; 63% for reader 2), and Gruen zone 9 (75% for reader 1; 50% for reader 2). Over time, there was a substantial decrease in periosteal edema around the femoral shaft: 24 months after the surgery, periosteal edema persisted only in 1 patient in Gruen zones 5 and 10 (13%, respectively for both readers) as well as in Gruen zone 2 (13% for reader 1) (Fig. 7). Never was any periosteal edema present adjacent to the acetabular cup.

#### Periarticular soft tissue edema

**Fig. 8:**
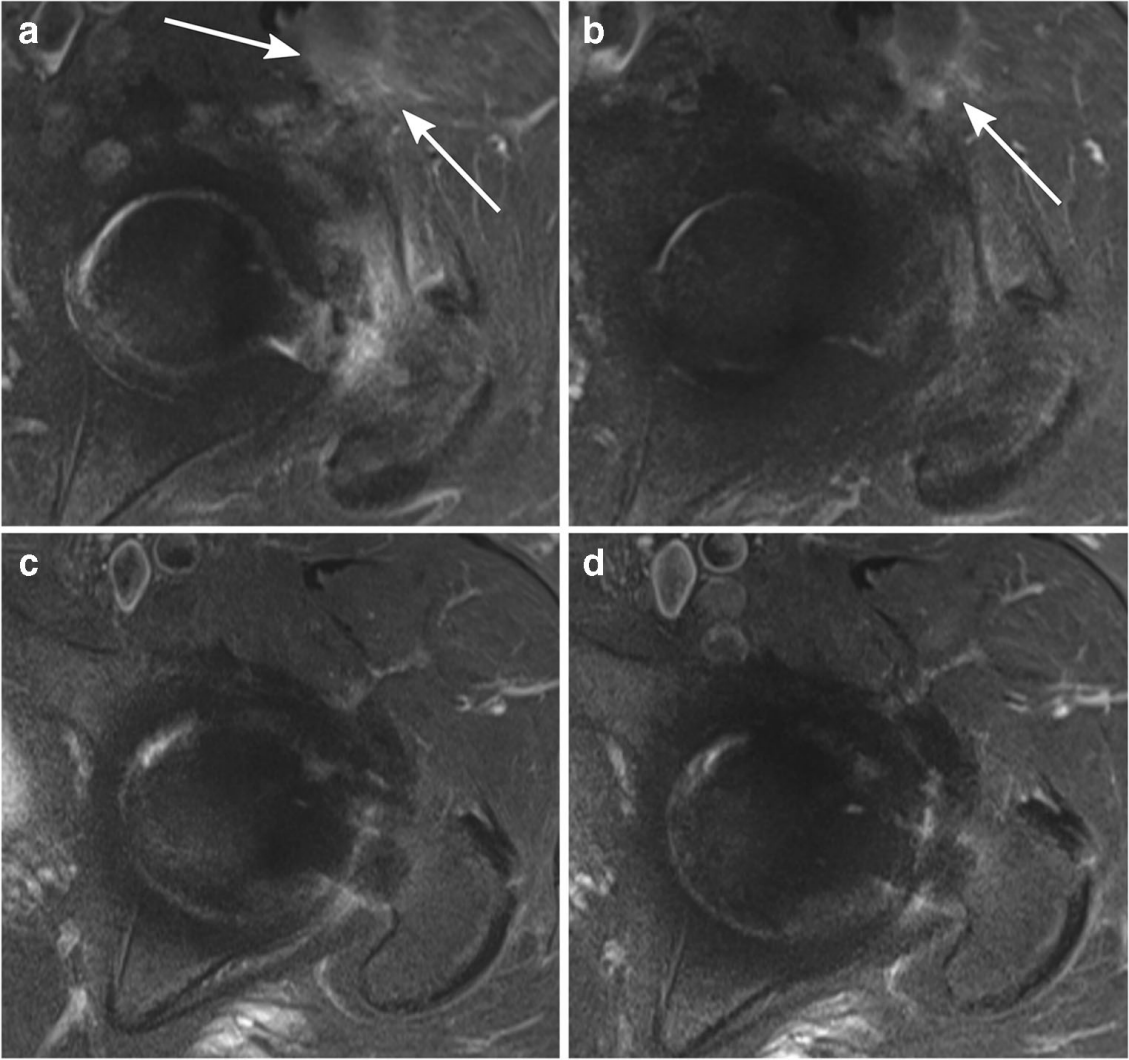
Periarticular soft tissue edema on axial STIR MRI. A 70-year-old asymptomatic woman with left-sided THA. Soft tissue edema (arrows) is present anteriorly along the surgical access route 3 months (**a**) and 6 months (**b**) after THA, which completely disappeared 12 months (**c**) and 24 months (**d**) after THA. *THA*, total hip arthroplasty

Periarticular soft tissue edema in the surgical access route (anterior) was present in all patients 3 months after surgery (100% for both readers) and almost all participants after 6 months (89% for reader 1; 100% for reader 2). No soft tissue edema in any location occurred after 12 or 24 months (Fig. 8).

#### Joint fluid

**Fig. 9:**
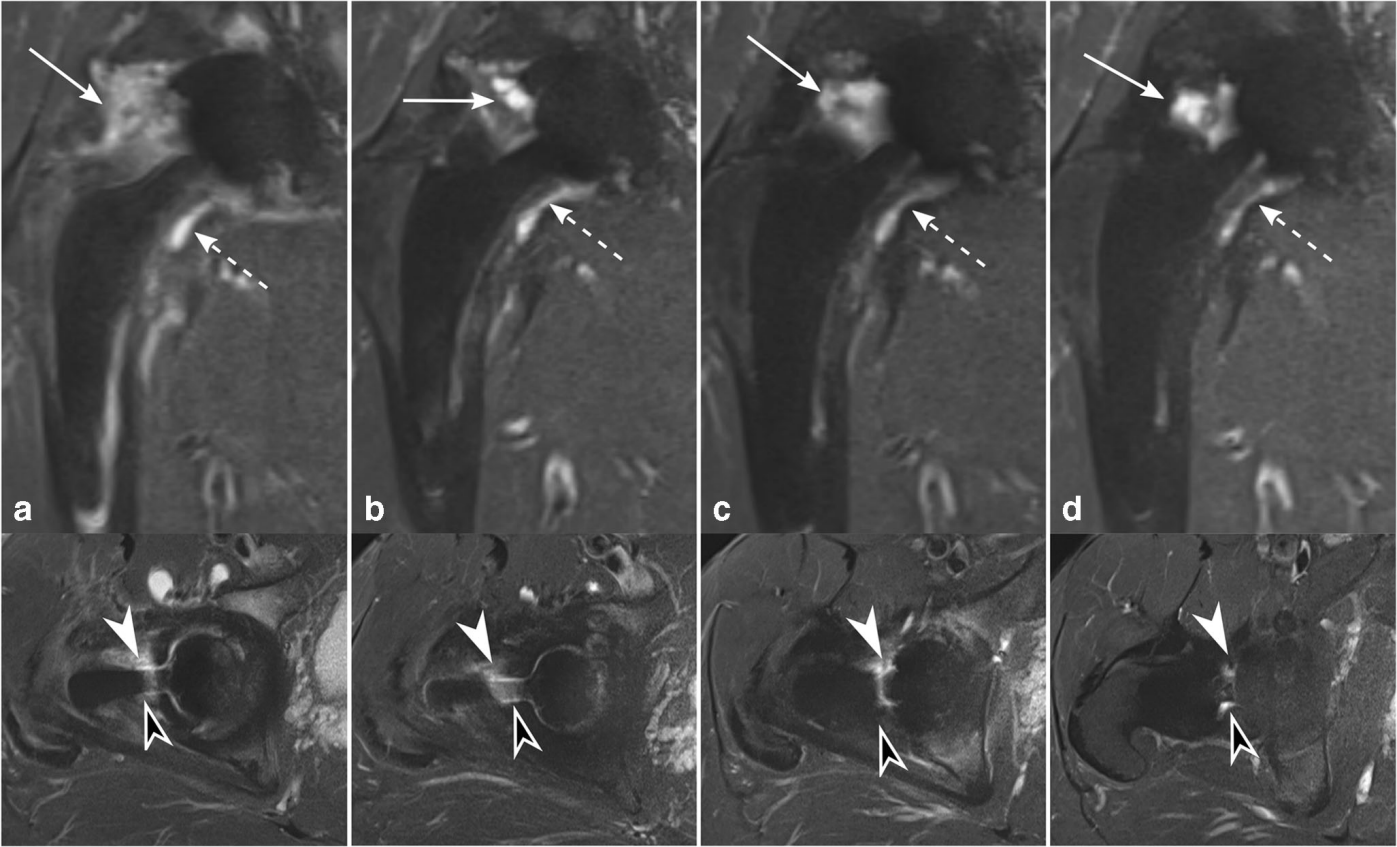
Joint effusion on coronal STIR CS-SEMAC (upper row) and on axial STIR (lower row) during the first two postoperative years (**a** = 3 months, **b** = 6 months, **c** = 12 months, and **d** = 24 months). A 51-year-old asymptomatic man with right-sided THA. Persisting joint effusion is clearly present laterally (arrows) with a maximum width of > 6 mm, whereas only a small amount of joint fluid (< 3 mm) is seen medially (dashed arrows). Anteriorly (white arrowhead) and posteriorly (black arrowhead), no clear joint effusion can be identified. *CS,* compressed sensing; *SEMAC*, slice encoding for metal artifact correction; *THA*, total hip arthroplasty

Detailed results regarding the location and quantity of joint fluid for both readers are listed in Table 2. After 3 months, several patients showed moderate to substantial amounts of joint fluid in the anterior (25% ≥ 3 mm for reader 1; 37.5% ≥ 3 mm for reader 2), medial (50% ≥ 3 mm for both readers), and lateral location (62.5% ≥ 3 mm for both readers), whereas no to mild joint effusion was present posteriorly during the whole follow-up period (100% < 3 mm for reader 1; 88–100% < 3 mm for reader 2) (Fig. 9). The amount of joint fluid in the anterior, medial, and lateral location decreased gradually from 3 to 24 months after THA, showing no to only mild joint effusion after 24 months in the majority of patients anteriorly and medially (100% < 3 mm for reader 1; 88–100% < 3 mm for reader 2) as well as laterally (75% < 3 mm for reader 1; 62.5% < 3 mm for reader 2).

**Table 2:** Amount of joint fluid, measured as the distance between the prosthetic head/neck and the joint capsule in 4 directions. For both readers, joint fluid was pronounced in the medial and lateral aspects. Over time, there was a slight decrease with persistent substantial joint fluid (> 6 mm) only in few patients in the lateral aspect after 12 and 24 months. *ant.*, anterior; *post.*, posterior; *med.*, medial; *lat.*, lateral; *mo*, months

Joint fluid		Reader 1				Reader 2			
		3 mo (%)	6 mo (%)	12 mo (%)	24 mo (%)	3 mo (%)	6 mo (%)	12 mo (%)	24 mo (%)
ant.	< 3 mm	75	89	100	100	62.5	89	100	100
	3–6 mm	12.5	11	0	0	12.5	11	0	0
	> 6 mm	*12.5*	0	0	0	*25*	0	0	0
post.	< 3 mm	100	100	100	100	87.5	100	100	100
	3–6 mm	0	0	0	0	12.5	0	0	0
	> 6 mm	0	0	0	0	0	0	0	0
med.	< 3 mm	50	67	100	100	50	67	100	88
	3–6 mm	25	22	0	0	25	22	0	12
	> 6 mm	*25*	*11*	0	0	*25*	*11*	0	0
lat.	< 3 mm	37.5	78	78	75	37.5	78	56	62.5
	3–6 mm	50	11	11	12.5	50	11	22	25
	> 6 mm	*12.5*	*11*	*11*	*12.5*	*12.5*	*11*	*22*	*12.5*

#### Gluteal tendons

Both the gluteus medius and minimus tendon were intact in nine of nine patients (100%) during the 24-month follow-up.

#### Other findings

No extraarticular fluid collection was present at any time.

## Discussion

The purpose of this prospective longitudinal study was to show the natural evolution of normal periprosthetic MRI findings in the first 2 years after THA in asymptomatic individuals. To our knowledge, this is the first prospective study in the scientific literature to address this issue.

BME after THA may occur due not only to intraoperative reaming and compaction techniques but also from pathologic conditions such as mechanical stress reaction, implant loosening, or infection [13, 22, 24, 32, 33]. Compared to a retrospective cross-sectional study [24], our prospective study gives incremental insight regarding the evolution of BME over time. In our cohort, BME occurred frequently and diffusely around the femoral stem as well as centrally at the acetabular cup 3 and 6 months after surgery. There was a substantial decrease in BME over time, persisting up to 24 months after THA around the upper lateral and medial femoral stem (Gruen zones 1 and 7) in some cases. This persistent BME up to 24 months after surgery in asymptomatic individuals can be a possible pitfall in MRI interpretation and should not be overcalled as unequivocal pathology, in particular, if it affects only single and non-adjoining Gruen zones. Importantly, BME at the inferomedial portion of the cup rarely occurred 3 months after surgery and never later than that, so any BME seen at this location 6 months or longer after surgery is suspicious of pathology.

Uncemented THA are designed with roughened or coated surfaces to facilitate osseous ingrowth [3]. However, a frequent finding in uncemented THA is the development of a fibrous membrane which can be considered a meta-stable status and should be followed for progression to implant loosening [1, 6, 22, 34]. We defined “ periprosthetic bone resorption” as a well-demarcated linear band between the implant surface and the host bone, independent of its thickness [24]. This feature was occasionally seen adjacent to the lateral and anterior shoulder of the femoral stem (Gruen zones 1 and 8) in the second postoperative year; however, the maximum width was 2 mm, which should not be considered a sign of loosening [1].

Periosteal edema can be a manifestation of osseous stress reaction or periprosthetic infection [1, 22]. However, it has been shown that periosteal edema may occur in asymptomatic individuals 12 months after THA, although less commonly than in symptomatic individuals [24]. Beyond that, our data illustrate the chronological evolution of periosteal edema, being very common 3 months after surgery in the anterior, lateral, and medial femoral shaft with a gradual decrease over the first 2 postoperative years, rarely persisting up to 24 months. Interestingly, periosteal edema—similar to the BME pattern—only occurred in non-adjacent Gruen zones after 24 months. There was no periosteal edema at the acetabulum, which may be attributed to the difference in force transmission between the femoral and acetabular side during hip arthroplasty—in particular using an anterior approach, which may put more mechanical stress on the femoral shaft [35, 36], therefore potentially causing longer persistent periosteal edema.

Periarticular soft tissue edema is a non-specific finding. For example, it may indicate a periprosthetic infection [22]. Our data imply that soft tissue edema in the surgical access route is a constant feature in the first 6 months, but never occurred in the second postoperative year.

Joint effusion is also a non-specific finding, caused e.g. by periprosthetic infection or wear-induced synovitis [1, 22] but may also be seen in asymptomatic individuals [24]. We found substantial joint effusion most commonly in the medial and lateral aspect 3 months after THA, decreasing with time after surgery. However, substantial joint effusion may be seen as late as 24 months after surgery in some asymptomatic individuals in the lateral aspect and therefore, these imaging findings imply careful interpretation. During the 24-month follow-up, both the gluteus medius and minimus tendons were intact in all patients, as can be expected when using an anterior surgical approach, accessing the hip joint between the rectus femoris and tensor fasciae latae muscle.

There are limitations to our study. First, the small cohort size (*n* = 9 patients) may limit the generalizability of our findings. However, four consecutive MRI examinations were available for each included subject and substantial to almost perfect inter-reader agreement represents plausible validity of the detected imaging findings. Second, this study was limited to primary uncemented THA with three different titanium-based systems. Therefore, our observations may not apply to other scenarios such as complex surgeries, revision surgeries, or cemented prostheses. Third, bone quality was not systematically documented, but may potentially affect the MRI findings in the periprosthetic bone after hip arthroplasty. Despite these limitations, we strongly believe that our data may serve as a standard reference regarding the evolution of normal postoperative MRI findings, which is crucial to be aware of in order to correctly evaluate postoperative complications and not to misinterpret actual normal findings.

In conclusion, BME and periosteal edema along the femoral stem are a frequent finding in asymptomatic patients within the first 6 months after THA, decreasing substantially in the following period. However, BME and periosteal edema may persist in a few non-adjoining Gruen zones in a few asymptomatic individuals up to 24 months and should not be mistaken as unequivocal pathology. The occurrence of periarticular soft tissue edema as late as 12 months after surgery should be considered an abnormal finding and necessitates further diagnostic workup, e.g., to exclude periprosthetic infection. A thin (≤ 2 mm) linear well-demarcated osseous resorption adjacent to the proximal and to a lesser degree adjacent to the distal femoral stem can be seen in the second postoperative year after THA in asymptomatic patients.

## Abbreviations

BME: Bone marrow edema
CS: Compressed sensing
mo: Months
SEMAC: Slice encoding for metal artifact correction
STIR: Short τ inversion recovery
THA: Total hip arthroplasty
WOMAC: Western Ontario and McMaster Universities Arthritis Index
